# Prevalence and Risk Factors for Adrenal Insufficiency in Patients with Multiple Myeloma Receiving Long-Term Chemotherapy including Corticosteroids: A Retrospective Cohort Study

**DOI:** 10.1155/2021/2330417

**Published:** 2021-12-13

**Authors:** Jee Hee Yoon, Seo-Yeon Ahn, Sung-Hoon Jung, Je-Jung Lee, Wonsuk Choi, Ji Yong Park, A Ram Hong, Hee Kyung Kim, Ho-Cheol Kang

**Affiliations:** Department of Internal Medicine, Chonnam National University Hwasun Hospital, 322 Seoyang-ro, Hwasun, Jeollanam-do 519-763, Republic of Korea

## Abstract

Multiple myeloma (MM) is the second most common hematologic malignancy and requires long-term and high-dose corticosteroid-based chemotherapy. The aim of this study was to investigate the prevalence and clinical predictors of corticosteroid-associated adrenal insufficiency (AI) in patients with MM receiving long-term chemotherapy. This retrospective study included patients with MM who were administered corticosteroid-based chemotherapy and underwent a rapid adrenocorticotropic hormone (ACTH) stimulation test between 2005 and 2018. AI was determined by a peak cortisol value < 18 *μ*g/dL after ACTH stimulation. Demographic, clinical, and laboratory parameters were evaluated, and the prevalence and clinical risk factors of AI were examined. Of 282 patients with MM who received corticosteroid-based chemotherapy, 142 patients (50.4%) were classified as having AI. There were no differences in age, sex, body mass index, comorbidities, and laboratory findings, including serum sodium levels between the AI and no-AI groups. In univariate analysis, the cumulative dose of corticosteroid (odds ratio (OR) = 0.99, 95% confidence interval (CI) 0.98–0.99; *P* = 0.020) and megestrol acetate use (OR = 2.63, 95% CI 1.48–4.67; *P* = 0.001) were associated with the occurrence of AI. Cumulative duration and cumulative dose per duration of corticosteroid use were not associated with the occurrence of AI. However, in the multivariate analysis, only megestrol acetate use was associated with an increased risk of AI (OR = 2.54, 95% CI 1.41–4.60; *P* = 0.002). Approximately 95.8% of patients with AI had suspicious symptoms or signs of AI. Although clinical symptoms and signs are usually nonspecific, symptomatic patients with MM receiving long-term corticosteroid therapy have sufficient potential for developing AI, particularly when receiving megestrol acetate. These findings can help alert clinicians to consider adrenal suppression following corticosteroid-based chemotherapy in patients with MM.

## 1. Introduction

Corticosteroid therapy has been used in malignant diseases because of its anticancer efficacy; however, it is associated with numerous adverse events [1, 2]. Chronic corticosteroid therapy is the most common cause of adrenal insufficiency (AI), which occurs due to inhibition of the hypothalamic-pituitary-adrenal (HPA) axis through a negative feedback mechanism after discontinuation of exogenous steroids [3, 4]. Although higher doses and long-term corticosteroid therapy may be risk factors for developing AI, previous reports have reported inconsistent results regarding this.

Multiple myeloma (MM) is caused by the proliferation of clonal plasma cells in the bone and bone marrow being the second most common hematological malignancy. In the past, induction therapy with alkylating agents and corticosteroids or high-dose chemotherapy followed by autologous hematopoietic stem cell transplantation were the main treatments for MM. In the last decade, the treatment of MM has evolved with the development of more effective drugs, including proteasome inhibitors, immunomodulatory drugs, monoclonal antibodies, and histone deacetylase inhibitors. However, each regimen is enhanced in combination with dexamethasone [5]. Consequently, the majority of patients with MM are exposed to long-term and high-dose corticosteroids and are thus prone to developing AI.

AI is potentially life-threatening, and corticosteroid replacement is essential to prevent adrenal crisis, particularly in acute stressful conditions such as trauma, surgery, and severe infection, which are common among patients receiving chemotherapy [6–8]. Therefore, early diagnosis of AI and proper corticosteroid replacement are important strategies to reduce the risk of mortality in these patients. However, to date, epidemiological data on AI in patients with MM have been sparse. In this context, we aimed to evaluate the prevalence and clinical predictors of AI in patients with MM receiving long-term chemotherapy.

## 2. Materials and Methods

### 2.1. Study Population

This retrospective study was conducted at Chonnam National University Hwasun Hospital, a tertiary referral cancer center in South Korea. The inclusion criteria were patients who received chemotherapy for newly diagnosed MM and underwent a rapid adrenocorticotropic hormone (ACTH) stimulation test during follow-up between January 2005 and December 2018. Among the 414 eligible patients, six patients who had received corticosteroid treatment or had preexisting AI before chemotherapy were excluded. Thirteen patients who did not receive at least two cycles of corticosteroid-based chemotherapy were also excluded. None of the patients took estrogen-containing medications. More than 90% of serum circulating cortisol is present in protein-bound form (i.e., cortisol binding globulin or albumin). Hence, in conditions with reduced binding proteins, such as hypoalbuminemia, cosyntropin-stimulated serum total cortisol concentrations may be measured lower than actual values, leading to false-positive AI results. Therefore, we further excluded 113 patients who had hypoalbuminemia at the time of performing the rapid ACTH stimulation test [9]. Finally, 282 patients with MM were included in the analysis.

This study was approved by the Institutional Review Board of Chonnam National University Hwasun Hospital (IRB No. CNUHH-2021-079) and was conducted in accordance with the guidelines of the Declaration of Helsinki. The need for informed consent was waived because of the retrospective design of the study.

### 2.2. Demographic Assessments and Corticosteroid Treatment

Clinical characteristics of patients, including age, sex, body mass index (BMI), and prevalence of comorbidities including hypertension, type 2 diabetes mellitus (T2DM), cardiovascular disease (CVD), thromboembolism, and chronic kidney disease (CKD), were evaluated based on the medical records. AI-associated clinical symptoms and signs, including general weakness, nausea, vomiting, anorexia, dizziness, and hypotension, were also retrospectively examined. The duration of follow-up was defined as the time interval from the initiation of chemotherapy to the rapid ACTH stimulation test.

We collected data on the type, dose, and duration of corticosteroid treatment during the follow-up period. To adjust for differences in corticosteroid potency among treatment regimens, we calculated the equivalent corticosteroid dose of prednisolone as the standard reference. Approximate equivalent dose for 20 mg of hydrocortisone was 5 mg of prednisolone, 4 mg of methylprednisolone, and 0.75 mg of dexamethasone [10]. The prevalence of megestrol acetate use was also evaluated in the last chemotherapy regimen. We included all patients who were prescribed megestrol acetate, regardless of the dose and duration.

### 2.3. Rapid ACTH Stimulation Test and Biochemical Measurements

The rapid ACTH stimulation test with 250 *μ*g of synthetic ACTH (Synacthen; Novartis, Basel, Switzerland) was used for the diagnosis of AI, regardless of associated symptoms or signs of AI. This test was started in the morning between 8 and 9 a.m., and 250 *μ*g of Synacthen was freshly prepared. After the collection of blood samples for serum cortisol and plasma ACTH at baseline, synthetic ACTH was administered intravenously. Additional blood samples for serum cortisol levels were collected at 30 min and 60 min after ACTH administration. The peak cortisol value after ACTH injection of less than 18 *μ*g/dL (500 nmol/L) was defined as AI [11]. Among 219 patients on corticosteroid therapy at the time of rapid ACTH stimulation test, 64 patients (29.2%) received prednisolone for 3–4 days a month with a median monthly dose of 75.0 mg (60.0–100.0) and 155 patients (70.8%) received dexamethasone therapy weekly, biweekly, or monthly with a median monthly dose of 37.0 mg (20.0–80.0). Temporal stopping of corticosteroid use is generally recommended for adrenal function assessment: 24 h for hydrocortisone and at least 48–72 h for prednisone, prednisolone, and dexamethasone before cosyntropin-stimulation test [12]. In the present study, the rapid ACTH stimulation test was performed at least 48 h after the last administration of corticosteroids, considering the biological half-life of exogenous corticosteroids. The mean time interval between the last dose of corticosteroid therapy and rapid ACTH stimulation test was 10.9 ± 13.5 months in 63 patients who had been on chemotherapy off status.

Thyroid function was evaluated in patients with suspicious symptoms such as hyponatremia or generalized edema. Otherwise, no other endocrine hormone evaluations were performed.

Serum cortisol levels were measured by radioimmunoassay using RIAZENco CORTISOL (R-JG-100, ZenTech, Liege, Belgium), and plasma ACTH was measured by immunoradiometric assay using ELISA-ACTH (Cisbio Bioassays International, Codolet, France). Estimated glomerular filtration rate (eGFR) was calculated using the Modification of Diet in Renal Disease equation (mL/min/1.73 m^2^) [13].

### 2.4. Statistical Analysis

Data are expressed as mean ± standard deviation or median (interquartile range) or *n* (%). Continuous variables were analyzed using Student's *t*-test or Mann-Whitney test if indicated, and categorical variables were analyzed using the chi-square test. The predictive factors for AI were obtained using univariate and multivariate logistic regression analyses. Risk is expressed as odds ratios (ORs) and 95% confidence intervals (CIs). All statistical analyses were performed using SPSS Statistics version 25 (IBM, Armonk, NY, USA). Statistical significance was set at *P* < 0.05.

## 3. Results

### 3.1. Baseline Characteristics of the Study Population

Of the 282 patients receiving corticosteroid-included chemotherapy for MM, 142 (50.4%) had AI (Table 1). There were no clinically or biochemically suspected patients with primary AI in the present study. In patients with AI, serum cortisol levels were 4.4 ± 3.2 *μ*g/dL at baseline, 9.9 ± 4.1 *μ*g/dL at 30 min, and 11.8 ± 4.5 *μ*g/dL at 60 min after ACTH stimulation. The mean time interval between the last corticosteroid administration and the rapid ACTH stimulation test was 100.4 ± 255.4 days in the no-AI group and 92.6 ± 218.4 days in the AI group (*P* = 0.781). There were no significant differences with regard to age, sex, BMI, and prevalence of comorbidities including hypertension, T2DM, CVD, CKD, and thromboembolism between the no-AI and AI groups (all *P* > 0.05). Biochemical parameters, including serum sodium, albumin, creatinine, calcium, aspartate aminotransferase, alanine aminotransferase, and eGFR levels, did not differ between the no-AI and AI groups (all *P* > 0.05).

### 3.2. Relationship between Corticosteroid Treatment and the Occurrence of AI

The median duration of follow-up was 27.0 months (interquartile range 10.0–59.8) in the no-AI group and 22.0 months (interquartile range 7.0–45.0) in the AI group (*P* = 0.076; Table 2). Approximately 75.0% and 80.3% of patients in the no-AI and AI groups, respectively, received chemotherapy at the time of the rapid ACTH stimulation test (*P* = 0.287). The mean number of changes in chemotherapy regimen was 2.1 ± 1.2 in the no-AI group and 2.0 ± 1.3 in the AI group (*P* = 0.497). There were no differences in the type of corticosteroid administered between the no-AI and AI groups (*P* = 0.367). The median cumulative duration of corticosteroid use did not differ between the no-AI and AI groups (58.5 days (interquartile range 21.3–136.5) vs. 47.5 days (interquartile range 24.0–102.0); *P* = 0.135). However, the median cumulative dose of corticosteroids was significantly lower in the AI group than in the no-AI group (25632 mg (interquartile range 11333–50712) vs. 28834 mg (interquartile range 12511–62262); *P* = 0.013). The median cumulative dose per duration of corticosteroid use was 525.9 mg/day (interquartile range 326.7–766.5) in the no-AI group and 456.2 mg/day (interquartile range 310.7–763.1) in the AI group (*P* = 0.803). Interestingly, the AI group showed a higher prevalence of megestrol acetate use than the no-AI group (33.1% vs. 15.7%, *P* = 0.001).

We separately analyzed the relationship between corticosteroid use and the occurrence of AI according to megestrol acetate use (Table 3). In megestrol acetate users, AI was associated with lower cumulative dose and duration of corticosteroids (*P* = 0.016 and *P* = 0.038, respectively), however, not with cumulative dose per duration of corticosteroid use (*P* = 0.549), while in megestrol acetate nonusers, no significant associations were observed between corticosteroid use and AI.

After the diagnosis of AI, steroid replacement was started with hydrocortisone of 10–20 mg or equivalent dose of other steroids, and adrenal crisis was observed in three patients during the observational period.

### 3.3. Univariate and Multivariate Analysis for Predicting AI

In the univariate analysis, age, sex, and BMI were not associated with AI. In addition, cumulative duration of corticosteroid use and cumulative dose per duration of corticosteroid use did not differ between the no-AI and AI groups (Table 4). Cumulative dose of corticosteroid use (OR = 0.99, 95% CI 0.98–0.99; *P* = 0.020) and megestrol acetate use (OR = 2.63, 95% CI 1.48–4.67; *P* = 0.001) were significantly associated with AI. However, the statistical significance only remained for megestrol acetate use in the multivariate analysis (OR = 2.54, 95% CI 1.41–4.60, *P* = 0.002).

### 3.4. Clinical Symptoms and Signs Associated with AI

The AI group had a higher frequency of suspicious symptoms and signs of AI than the no-AI group (95.8% vs. 87.1%; *P* = 0.009). Of these, general weakness was the most frequently reported symptom, followed by dizziness, hypotension, gastrointestinal problems, and anorexia. Notably, six of the 142 patients with AI had no obvious symptoms or signs suggesting AI (Table 5). Serum peak cortisol level was associated with the presence of AI-related symptoms or signs (OR = 0.95, 95% CI 0.92–0.99; *P* = 0.013).

## 4. Discussion

We demonstrated that half of the patients with MM who received corticosteroid-based long-term chemotherapy had AI. Patients with AI were more frequently treated with megestrol acetate compared to those without AI. However, cumulative dose, duration, and type of corticosteroid use were not associated with AI. Meanwhile, most patients diagnosed with AI presented with suspicious symptoms and signs of AI during the rapid ACTH stimulation test. These findings indicate that the clinical suspicion of AI is crucial for detecting AI regardless of the underlying corticosteroid treatment regimen.

Recurrent exposure to exogenous corticosteroids can suppress the HPA axis, thereby eliciting AI [14, 15]. Corticosteroid-induced AI has a wide prevalence rate of 14–63% across studies [15, 16]. Most of the previous studies examined the risk of AI in patients receiving oral and inhaled daily corticosteroid therapy. Few studies have investigated AI caused by high-dose cyclic corticosteroid therapy in patients receiving chemotherapy for hematologic malignancies (i.e., lymphoma and leukemia) [17–20]. The prevalence of AI in these diseases was reported to be 23–40% over 14–90 days of follow-up, with most patients being children. In lymphoma and leukemia, high-dose corticosteroids are usually administered over a relatively short period of 4–6 cycles. In contrast, MM is a chronic disease that requires high-dose and cyclic but long-term corticosteroid treatment, which further increases the risk of AI. However, there are limited data on the epidemiology of AI in patients with MM. Ng et al. reported that 40% (18/45) of patients showed adrenal suppression after 3 months of a dexamethasone-based regimen [21]. Ahn et al. observed that 48.3% (28/58) of hospitalized MM patients had AI after 12.7 months of corticosteroid-included chemotherapy [22]. In the present study, the prevalence of AI was 50.4%, which is similar to or slightly higher than previous results for MM as well as other hematologic malignancies. To the best of our knowledge, this is the largest study to evaluate the epidemiology of AI in patients with MM.

The underlying pathophysiological mechanism and risk factors for the occurrence of corticosteroid-related AI remain unclear. Previous studies have reported heterogeneous results with respect to the type, dose, duration, and routes of corticosteroid administration for developing AI [16]. This indicates the influence of individual variations on the susceptibility of HPA suppression by exogenous corticosteroids. One study showed that corticosteroid dose and duration were not associated with AI [23], while other studies did [24, 25]. In our study, duration, cumulative dose, and type of corticosteroid treatment were not associated with the occurrence of AI. One of the explanations for this is the time interval from initiation of chemotherapy to the evaluation of AI. The median time interval between the initiation of chemotherapy and the rapid ACTH stimulation test was approximately 3 years, which is considerably longer than that in previous studies [21, 22]. This suggests that all patients had already been exposed to high-dose corticosteroid treatment, and the impact of corticosteroids on AI risk may have been underestimated.

In the present study, AI was more frequently observed in patients taking megestrol acetate during the last chemotherapy regimen. Megestrol acetate is a synthetic progestational agent that has been used for anorexia and cachexia in patients with malignancies, including MM [26]. Because megestrol acetate has corticosteroid-like activity, it can suppress the HPA axis, thereby resulting in AI [26, 27]. A multicenter prospective study in Korea revealed a higher incidence of AI in patients with cancer treated with antiemetic dexamethasone and megestrol acetate than in those treated with antiemetic dexamethasone alone [28]. Given that patients with symptomatic AI may be treated more often with megestrol acetate, it was difficult to determine the causal relationship between megestrol acetate and AI in our study. However, more careful attention to AI is needed for patients with MM who are treated with long-term steroid therapy and megestrol acetate together.

Clinical symptoms caused by chronic low cortisol levels, such as fatigue, nausea, vomiting, anorexia, and general weakness, are nonspecific [4] and similar to common symptoms experienced by patients with chronic illness, particularly advanced cancer. Therefore, suspicious symptoms of AI can be easily overlooked. However, if the diagnosis is delayed, the lack of an appropriate cortisol response to stressful conditions may lead to life-threatening adrenal crisis [29]. In our study, of the 258 patients with suspicious symptoms or signs of AI, 136 (52.8%) had AI, indicating a difficulty in distinguishing AI from no-AI without a definite hormonal test. In addition, six patients were determined to have AI without any clinical symptoms or signs. Considering the cost-effectiveness, routine assessment of adrenal function is not required. However, high clinical suspicion with adequate hormonal evaluation may help detect AI in patients with MM receiving long-term corticosteroid-based chemotherapy, particularly if the patient has symptoms and signs relevant to AI.

We acknowledge that there are several limitations to be addressed in this study. First, due to its retrospective design, a rapid ACTH stimulation test was not performed in all patients with MM either before chemotherapy or during follow-up, leading to overestimation of the prevalence of AI. Second, since most patients (77.7%) continued corticosteroid-based chemotherapy, we could not conclude whether adrenal suppression recovered to normal cortisol response or persisted. Only 10 patients underwent a follow-up rapid ACTH stimulation test after discontinuation of corticosteroid-based chemotherapy. Peak serum cortisol levels increased in all patients, however, still below 18 *μ*g/dL (500 nmol/L). Third, recent studies have suggested new lower serum cortisol cutoff value to reduce overdiagnosis of AI using specific monoclonal antibody immunoassays or liquid chromatography–tandem mass spectrometry methods [30]. However, in this study, serum cortisol was measured using conventional polyclonal antibody assay. In our study, 115 patients (40.8%) met the AI criteria when AI was defined as the peak serum cortisol value after ACTH stimulation of less than 16.3 *μ*g/dL (450 nmol/L). Future studies are needed to reveal better assay methods and cutoff values to reduce false positives in AI diagnosis. Lastly, megestrol acetate had a cortisol suppressive effect, which could affect the analysis of the association between AI and steroid-related factors such as dose, type, and duration. In the present study, when we analyzed the association between corticosteroid use and AI according to megestrol acetate use, high dose or duration of corticosteroid use was not associated with the development of AI regardless of megestrol acetate use. Further, patients with AI tend to receive megestrol acetate for improving anorexia and well-being. Thus, in the setting of the retrospective nature of a study such as ours, it is difficult to establish a causal relationship between megestrol acetate and AI, and caution is required in interpretation.

Despite these limitations, our study is valuable as it determines the real-world incidence and clinical risk factors of AI in patients with MM based on a large retrospective cohort. The present findings can alert clinicians to consider adrenal suppression following corticosteroid-based chemotherapy in patients with MM.

## 5. Conclusion

In summary, MM patients treated with long-term cyclic high-dose corticosteroids have a significant prevalence of corticosteroid-induced AI. Administration of megestrol acetate is associated with an increased risk of AI. Regarding corticosteroid administration, the dose, type, and duration of corticosteroid therapy did not predict the occurrence of AI. While most of the symptoms associated with AI are nonspecific and easily observed in malignant patients, high clinical suspicion of AI is important to adequately detect AI and prevent AI-related fatal outcomes.

## Figures and Tables

**Table 1:** Baseline characteristics of patients with multiple myeloma according to the presence of adrenal insufficiency.

Variables	Overall (*n* = 282)	No-AI (*n* = 140)	AI (*n* = 142)	*P* value
Age (years)	66.6 ± 8.8	65.7 ± 9.5	67.5 ± 8.0	0.076
Sex (male)	148 (52.5)	75 (53.6)	73 (51.4)	0.716
BMI (kg/m^2^)	24.3 ± 3.6	24.0 ± 3.5	24.5 ± 3.8	0.333
Comorbidities				
Hypertension	135 (47.9)	73 (52.1)	62 (43.7)	0.154
Diabetes	91 (32.3)	46 (32.9)	45 (31.7)	0.834
CVD	33 (11.7)	18 (12.9)	15 (10.6)	0.549
CKD	115 (40.8)	58 (41.4)	57 (40.1)	0.826
Thromboembolism	25 (8.9)	11 (7.9)	14 (9.9)	0.554
Laboratory findings				
WBC (×10^3/^*μ*L)	4.8 ± 2.6	4.8 ± 2.8	4.8 ± 2.3	0.792
Hemoglobin (g/dL)	11.0 ± 1.5	10.9 ± 1.6	11.0 ± 1.5	0.943
PLT (× 10^3/^*μ*L)	144.5 ± 79.6	147.1 ± 81.2	141.9 ± 78.1	0.587
AST (IU/L)	23.7 ± 9.6	23.0 ± 9.3	24.4 ± 9.8	0.223
ALT (IU/L)	22.3 ± 13.5	21.0 ± 12.0	23.6 ± 14.8	0.106
Creatinine (mg/dL)	1.28 ± 1.10	1.25 ± 1.06	1.32 ± 1.15	0.565
eGFR (mL/min/1.73 m^2^)	68.0 ± 28.5	70.2 ± 30.1	65.9 ± 26.7	0.199
Total protein (g/dL)	6.4 ± 0.5	6.4 ± 1.1	6.3 ± 0.8	0.672
Albumin (g/dL)	3.8 ± 0.5	3.8 ± 0.5	3.8 ± 0.4	0.987
Sodium (mEq/L)	140.8 ± 5.5	140.4 ± 6.3	141.3 ± 4.5	0.176
Calcium (ionized) (mEq/L)	2.34 ± 0.17	2.35 ± 0.15	2.33 ± 0.18	0.765
Rapid ACTH stimulation test				
ACTH (basal) (pg/mL)	37.0 (19.0–57.0)	43.5 (27.0–66.8)	26.9 (15.8–51.3)	<0.001
Cortisol (basal) (*μ*g/dL)	8.0 ± 6.0	11.7 ± 5.9	4.4 ± 3.2	<0.001
Cortisol (30 min) (*μ*g/dL)	15.6 ± 7.5	21.4 ± 5.6	9.9 ± 4.1	<0.001
Cortisol (60 min) (*μ*g/dL)	18.6 ± 10.6	25.5 ± 10.6	11.8 ± 4.5	<0.001

Data are presented as mean ± standard deviation or median (interquartile range) or *n* (%). AI: adrenal insufficiency; BMI: body mass index; CVD: cardiovascular disease; CKD: chronic kidney disease; WBC: white blood cell count; PLT: platelet; AST: aspartate aminotransferase; ALT: alanine aminotransferase; eGFR: estimated glomerular filtration rate; ACTH: adrenocorticotropic hormone.

**Table 2:** Corticosteroid treatment during chemotherapy in patients with multiple myeloma according to the presence of adrenal insufficiency.

Variables	No-AI (*n* = 140)	AI (*n* = 142)	*P*
Duration of follow-up (months)	27.0 (10.0–59.8)	22.0 (7.0–45.0)	0.076
Number of changes in chemotherapy regimen	2.1 ± 1.2	2.0 ± 1.3	0.497
Type of corticosteroid			0.367
Prednisolone only	19 (13.6)	27 (19.0)	
Dexamethasone only	37 (26.4)	40 (28.2)	
Combination^a^	84 (60.0)	75 (52.8)	
Cumulative dose of corticosteroids (mg)^b^	28834 (12511–62262)	25632 (11333–50712)	0.013
Cumulative duration of corticosteroid use (days)	58.5 (21.3–136.5)	47.5 (24.0–102.0)	0.135
Cumulative dose/duration of corticosteroid use (mg/day)^b^	525.9 (326.7–766.5)	456.2 (310.7–763.1)	0.803
Megestrol acetate use in the last chemotherapy regimen	22 (15.7)	47 (33.1)	0.001

Data are presented as mean ± standard deviation or median (interquartile range) or *n* (%). ^a^Eleven patients who had a history of methylprednisolone use were included. ^b^Corticosteroid doses were expressed in prednisolone equivalents.

**Table:** (a) Megestrol acetate user (*n* = 69)

	No-AI (*n* = 22)	AI (*n* = 47)	*P*
Age (years)	67.6 ± 7.2	69.2 ± 7.8	0.420
Sex (male)	13 (59.1)	25 (53.2)	0.646
BMI (kg/m^2^)	23.1 ± 5.2	23.5 ± 5.9	0.675
Type of corticosteroid			0.335
Prednisolone only	4 (18.2)	14 (29.8)	
Dexamethasone only	12 (54.5)	23 (48.9)	
Combination	6 (27.3)	10 (21.3)	
Cumulative dose of corticosteroids (mg)^a,b^	41166 (11283–65049)	17088 (7476–33108)	0.016
Cumulative duration of corticosteroid use (days)^a^	63.0 (31.8–111.0)	36.0 (18.0–64.0)	0.038
Cumulative dose/duration of corticosteroid use (mg/day)^a,b^	532.8 (280.0–741.3)	366.7 (278.9–700.5)	0.549

**Table:** (b) Megestrol acetate nonuser (*n* = 213)

	No-AI (*n* = 118)	AI (*n* = 95)	*P*
Age (years)	65.3 ± 9.8	66.7 ± 8.0	0.266
Sex (male)	62 (52.5)	48 (50.5)	0.770
BMI (kg/m^2^)	24.3 ± 3.6	24.6 ± 3.8	0.093
Type of corticosteroid			0.617
Prednisolone only	15 (12.7)	13 (13.7)	
Dexamethasone only	72 (61.0)	52 (54.7)	
Combination	31 (26.3)	30 (31.6)	
Cumulative dose of corticosteroids (mg)^b^	28132 (12981–61840)	33308 (12800–58035)	0.199
Cumulative duration of corticosteroid use (days)	55.5 (20.0–138.8)	60.0 (28.0–110.0)	0.811
Cumulative dose/duration of corticosteroid use (mg/day)^b^	519.9 (360.2–770.9)	504.0 (320.1–801.0)	0.772

Data are presented as mean ± standard deviation or median (interquartile range) or *n* (%). ^a^*P* values were generated by Mann-Whitney test. ^b^Corticosteroid doses were expressed in prednisolone equivalents.

**Table 4:** Clinical risk factors for adrenal insufficiency in patients with multiple myeloma.

	Univariate analysis			Multivariate analysis		
	OR	95% CI	*P*	OR	95% CI	*P*
Age	1.03	0.99–1.05	0.077	1.03	0.99–1.06	0.121
Sex	1.09	0.68–1.74	0.716	0.98	0.56–1.65	0.946
Body mass index	1.03	0.97–1.10	0.332	1.05	0.98–1.12	0.210
Cumulative dose of corticosteroids^a^	0.99	0.98–0.99	0.020	0.99	0.99–1.00	0.051
Cumulative duration of corticosteroid use	1.00	1.00–1.00	0.455	1.00	0.99–1.00	0.480
Cumulative dose/duration of corticosteroid use^a^	1.00	0.99–1.00	0.802	1.01	1.00–1.00	0.269
Megestrol acetate use in the last regimen	2.63	1.48–4.67	0.001	2.54	1.41–4.60	0.002

OR: odds ratio; CI: confidence interval. ^a^Corticosteroid doses were expressed in prednisolone equivalents.

**Table 5:** Subjective symptoms and signs of adrenal insufficiency in patients with multiple myeloma.

	No-AI (*n* = 140)	AI (*n* = 142)	*P* value
No suspicious symptoms and signs	18 (12.9)	6 (4.2)	0.009
Symptoms and signs	122 (87.1)	136 (95.8)	
General weakness	114 (81.4)	127 (89.5)	
Anorexia	2 (1.4)	1 (0.7)	
Nausea or vomiting	1 (0.7)	2 (1.4)	
Dizziness	4 (2.9)	3 (2.1)	
Hypotension	1 (0.7)	3 (2.1)	

Data are presented as *n* (%).

## Data Availability

The data used to support the findings of this study are available from the corresponding author upon request.

## References

[1] Charmandari E., Nicolaides N. C., Chrousos G. P. (2014). Adrenal insufficiency. *The Lancet*.

[2] van Staa T. P., Leufkens H. G., Abenhaim L., Begaud B., Zhang B., Cooper C. (2000). Use of oral corticosteroids in the United Kingdom. *QJM*.

[3] Oelkers W. (1996). Adrenal insufficiency. *The New England Journal of Medicine*.

[4] Arlt W., Allolio B. (2003). Adrenal insufficiency. *Lancet*.

[5] Röllig C., Knop S., Bornhäuser M. (2015). Multiple myeloma. *Lancet*.

[6] Podolak-Dawidziak M., Silber-Kasprzak D. (1976). Adrenal function in chronic myeloid leukemia. *Acta Haematologica Polonica*.

[7] Li W., Okwuwa I., Toledo-Frazzini K., Alhomosh A. (2013). Adrenal crisis in a patient with acute myeloid leukaemia. *BML Case Reports*.

[8] Iwasaku M., Tanaka S., Shinzawa M., Kawakami K. (2019). Impact of underlying chronic adrenal insufficiency on clinical course of hospitalized patients with adrenal crisis: a nationwide cohort study. *European Journal of Internal Medicine*.

[9] Bornstein S. R., Allolio B., Arlt W. (2016). Diagnosis and treatment of primary adrenal insufficiency: an Endocrine Society clinical practice guideline. *The Journal of Clinical Endocrinology and Metabolism*.

[10] Meikle A. W., Tyler F. H. (1977). Potency and duration of action of glucocorticoids: Effects of hydrocortisone, prednisone and dexamethasone on human pituitary-adrenal function. *The American Journal of Medicine*.

[11] Munro V., Elnenaei M., Doucette S., Clarke D. B., Imran S. A. (2018). The effect of time of day testing and utility of 30 and 60 minute cortisol values in the 250 mcg ACTH stimulation test. *Clinical Biochemistry*.

[12] Bancos I., Hahner S., Tomlinson J., Arlt W. (2015). Diagnosis and management of adrenal insufficiency. *The Lancet Diabetes and Endocrinology*.

[13] Levey A. S., Bosch J. P., Lewis J. B., Greene T., Rogers N., Roth D. (1999). A more accurate method to estimate glomerular filtration rate from serum creatinine: a new prediction equation. Modification of Diet in Renal Disease Study Group. *Annals of Internal Medicine*.

[14] Pazderska A., Pearce S. H. (2017). Adrenal insufficiency - recognition and management. *Clinical Medicine (London, England)*.

[15] Joseph R. M., Hunter A. L., Ray D. W., Dixon W. G. (2016). Systemic glucocorticoid therapy and adrenal insufficiency in adults: a systematic review. *Seminars in Arthritis and Rheumatism*.

[16] Broersen L. H., Pereira A. M., Jørgensen J. O. L., Dekkers O. M. (2015). Adrenal insufficiency in corticosteroids use: systematic review and meta-analysis. *The Journal of Clinical Endocrinology and Metabolism*.

[17] Owattanapanich W., Sirinvaravong S., Suphadirekkul K., Wannachalee T. (2018). Transient adrenal insufficiency in diffuse large B cell lymphoma patients after chemotherapy with short-course, high-dose corticosteroids. *Annals of Hematology*.

[18] Kuperman H., Damiani D., Chrousos G. P. (2001). Evaluation of the hypothalamic-pituitary-adrenal axis in children with leukemia before and after 6 weeks of high-dose glucocorticoid therapy. *The Journal of Clinical Endocrinology and Metabolism*.

[19] Einaudi S., Bertorello N., Masera N. (2008). Adrenal axis function after high-dose steroid therapy for childhood acute lymphoblastic leukemia. *Pediatric Blood & Cancer*.

[20] Rensen N., Gemke R. J., van Dalen E. C., Rotteveel J., Kaspers G. J., Cochrane Childhood Cancer Group (2017). Hypothalamic-pituitary-adrenal (HPA) axis suppression after treatment with glucocorticoid therapy for childhood acute lymphoblastic leukaemia. *Cochrane Database of Systematic Reviews*.

[21] Ng A. C., Kumar S. K., Russell S. J., Rajkumar S. V., Drake M. T. (2009). Dexamethasone and the risk for adrenal suppression in multiple myeloma. *Leukemia*.

[22] Ahn S. Y., Kim H. K., Kang H. C. (2021). Adrenal insufficiency in hospitalized patients with multiple myeloma. *Leukemia & Lymphoma*.

[23] Schlaghecke R., Kornely E., Santen R. T., Ridderskamp P. (1992). The effect of long-term glucocorticoid therapy on pituitary-adrenal responses to exogenous corticotropin-releasing hormone. *The New England Journal of Medicine*.

[24] Jamilloux Y., Liozon E., Pugnet G. (2013). Recovery of adrenal function after long-term glucocorticoid therapy for giant cell arteritis: a cohort study. *PLoS One*.

[25] Laugesen K., Petersen I., Sørensen H. T., Jørgensen J. O. L. (2019). Clinical indicators of adrenal insufficiency following discontinuation of oral glucocorticoid therapy: a Danish population-based self-controlled case series analysis. *PLoS One*.

[26] Leśniak W., Bała M., Jaeschke R., Krzakowski M. (2008). Effects of megestrol acetate in patients with cancer anorexia-cachexia syndrome--a systematic review and meta-analysis. *Polskie Archiwum Medycyny Wewnętrznej*.

[27] Ron I. G., Soyfer V., Goldray D., Inbar M. J., Weisman Y. (2002). A low-dose adrenocorticotropin test reveals impaired adrenal function in cancer patients receiving megestrol acetate therapy. *European Journal of Cancer*.

[28] Han H. S., Park J. C., Park S. Y. (2015). A prospective multicenter study evaluating secondary adrenal suppression after antiemetic dexamethasone therapy in cancer patients receiving chemotherapy: a Korean South West Oncology Group study. *The Oncologist*.

[29] Allolio B. (2015). Extensive expertise in ENDOCRINOLOGY: Adrenal crisis. *European journal of endocrinology*.

[30] Javorsky B. R., Raff H., Carroll T. B. (2021). New cutoffs for the biochemical diagnosis of adrenal insufficiency after ACTH stimulation using specific cortisol assays. *Journal of the Endocrine Society*.

